# In the same boat? An online group career counseling with a group of young adults in the time of COVID-19

**DOI:** 10.1007/s10775-021-09505-z

**Published:** 2021-10-07

**Authors:** S. Santilli, M. C. Ginevra, I. Di Maggio, S. Soresi, L. Nota

**Affiliations:** 1 University of Padua, Via Venezia, 14, 35100 Padua, Italy; 2 Department of Philosophy, Sociology, Education and Applied Psychology, University of Padua, Via Venezia, 14, 35100 Padua, Italy

**Keywords:** Online career counseling, COVID-19, Social Sustainability

## Abstract

An online group of career counseling for unemployed young adults during the COVID-19 pandemic was developed. Twelve participants were involved in online group career counseling intervention, based on the Life Design for an inclusive and sustainable future. Results indicated at post-test on increased scores on career adaptability, resilience, future orientation, and propensity to identify inclusive and sustainable actions for the future than pre-test. Overall, the online group career counseling intervention effectively promoted particular aspects of young adults' life design for an inclusive e-sustainable future.

## Résumé

**Dans le Même Bateau : Un Groupe de Conseil en Orientation Professionnelle En Ligne avec un Groupe de Jeunes Adultes à l'Époque du COVID-19** Un groupe de conseil en orientation professionnelle en ligne pour les jeunes adultes sans emploi pendant la pandémie de COVID-19 a été développé. Douze participant·e·s ont été impliqués dans une intervention de conseil en en orientation de groupe en ligne, basée sur le Life Design pour un avenir inclusif et durable. Les résultats indiquent que les scores sur l'adaptabilité de la carrière, la résilience, l'orientation future et la propension à identifier des actions inclusives et durables pour l'avenir ont augmenté au post-test par rapport au pré-test. Dans l'ensemble, l'intervention de conseil en orientation professionnelle de groupe en ligne a efficacement promu des aspects particuliers du Life Design chez jeunes adultes pour un avenir inclusif et durable.

## Abstract (de)

**Im selben Boot: Eine Online-Gruppen-Berufsberatung mit einer Gruppe junger Erwachsener in der Zeit von COVID-19** Es wurde eine Online-Berufsberatungsgruppe arbeitsloser junger Erwachsener während der COVID-19-Pandemie entwickelt. Zwölf Teilnehmer nahmen an dieser Online-Gruppen-Beratungsintervention teil, die auf dem Ansatz „Life Design” für eine integrative und nachhaltige Zukunft basierte. Die Ergebnisse zeigten, dass in der Nachbefragung die Werte für berufliche Anpassungsfähigkeit, Resilienz, Zukunftsorientierung und die Bereitschaft, integrative und nachhaltige Maßnahmen für die Zukunft zu identifizieren, höher waren als bei der Vorabbefragung. Insgesamt förderte die Online-Gruppen-Berufsberatung effektiv bestimmte Aspekte des Lebensentwurfs junger Erwachsener für eine integrative und nachhaltige Zukunft.

## Resumen

**En el mismo barco: un asesoramiento profesional grupal online con un grupo de adultos jóvenes en tiempos del COVID-19** Se desarrolló un grupo online de asesoramiento profesional para adultos jóvenes desempleados durante la pandemia de COVID-19. Doce participantes fueron involurados en una experiencia de asesoramiento profesional grupal online basada en el Life Design para un futuro inclusivo y sostenible. Los resultados del post-test de la experiencia indicaron un aumento significativo de las puntuaciones en adaptabilidad profesional, resiliencia, orientación futura y propensión a identificar acciones inclusivas y sostenibles para el futuro de las obtenidas antes de la aplicación de la prueba. En general, la intervención de asesoramiento profesional grupal online promovió efectivamente aspectos particulares del proyecto de vida de los adultos jóvenes para un futuro inclusivo y sostenible.

## Introduction

The COVID-19 pandemic is a health emergency and implies a significant crisis in the economy and the labor market that has an enormous impact on people and particularly young adults, globally. Even before the onset of the crisis, being a young adult placed in the labor market was an ongoing challenge due to a work environment already pervaded by crises and global challenges, such as increasing social injustice, ecological threats, and the flexibility of work contracts. This situation has led to a substantial increase in the level of discomfort toward future career planning in an increasing number of young adults who are grappling with hurdles and obstacles in the realization of their plans (Nota et al., 2020; Hite & McDonald, 2020; Pouyaud & Guichard, 2017).

However, the pandemic appears to be having a “devastating and disproportionate” impact on young adults’ employment: the most recent European-level data published in July 2020 (EUROSTAT, 2020) show an increase in the youth unemployment rate to 16.8%, with Italy, Sweden, Spain, and Greece exceeding 27%. The findings from the Global Survey on Youth and COVID-19 (International Labour Organization—ILO, 2020), involving over 12,000 responses from 112 countries between April and May 2020, revealed that 17.4% of young adults aged 18–29 years around the world had stopped working since the onset crisis, and indicated as primary reasons for job loss the end of the activities of the business they worked in for closing down, or a temporary job not renewed. Moreover, young adults in employment before the pandemic reported a 23% reduction in working hours and a lower income.

According to the ILO (2020), young adults constitute the principal victims of the pandemic’s social and economic consequences, and there is a risk that they will be scarred throughout their working lives—leading to the emergence of a “lockdown generation”. The COVID-19 crisis is expected to create more obstacles for young people in the job market: for young adults, the lack of jobs is likely to result in longer school-to-work transitions. Moreover, those already working are at risk of losing jobs amid the current wave of lay-offs and the collapse of businesses and start-ups (ILO, 2020).

Concerning the enormous pandemic disruption, especially for those who have lost their employment or are looking for a job, there is a growing interest in this topic in the field of vocational and career guidance, as reflected by a recent special issue (see Fouad, 2020). Suggestions for research on this phenomenon and its impact on workers and young adult job seekers are provided. In particular, Blustein et al. (2020), focusing on global unemployment, recommend “that research effort be constructed from the lived experiences of the individuals who are now out of work” (p. 3). They inspire scholars and practitioners to develop research and career interventions, including counseling strategies and systemic interventions, providing tangible support, especially for young people, during this crisis. These studies may improve the process of collective empowerment and critical consciousness development.

In light of this, we respond to this call proposing an online group career counseling intervention for young adults to design a decent work and life based on the Life Design for an inclusive and sustainable future during the COVID-19 pandemic in Italy. The intervention helps young people develop career adaptability, resilience, future orientation, and shift their thoughts from preoccupation with the present to reflections gradually focused on future intention to undertake professional activities that contribute to the realization of inclusive and sustainable social contexts. It also encourages thoughts about the future characterized by a sense of hope toward it and what they can do to manage it.

Specifically, the goal of the present study is to present the preliminary qualitative and quantitative analysis of its validity and effectiveness.

### Life designing for an inclusive, sustainable, and equitable future

The Life Design International Research Group has its foundation on social constructivism’s epistemology and acknowledges that career development is highly contextualized and individualized. It emphasizes the strong interconnection among the various life spheres, underlining that career development is to be intended as a dynamic interaction between a person and his/her environment, and all the life spheres that are relevant to that person have to be considered for the constructions of career and life projects (Nota & Rossier, 2015; Savickas et al., 2009).

According to the Life Design International Research Group, career interventions should focus on reflectivity, intended as a reflective project on the self and should consider the consequences of one’s own career decisions, intentionality, and action. This process involves translating intentions into actions to ensure that what is relevant to the individual will happen (Nota & Rossier, 2015; Di Maggio et al., 2019). Attention should also be paid to narratability, asking clients to produce micronarratives about how they have developed and constructed their self, identity, and career, promoting new meanings to their difficulties, thus triggering change emancipation processes (Savickas, 2015; Savickas et al., 2009). Emphasis should be given to the promotion of skills and resources to cope with the changes in the realm of work successfully. Resilience, positive future orientation and career adaptability are considered the Life Design International Research Group cornerstones (Nota & Rossier, 2015).

Recently, particular attention has been given to inclusion and sustainability, human rights, and social justice in career and life interventions for the construction and management of personal projects for decent work and a decent life for everyone, and especially for those who, nowadays, experience difficulties in the work environment, such as young adults (Guichard, 2018; Nota et al., 2020). In particular, because of the global threats that the socio-economic context is currently facing, it is essential to support people’s future design from an inclusive and sustainable perspective. In this sense, it is central to promote in young adults’ greater awareness about the processes that characterize the labor market and the social context, as well as the development of critical awareness, critical thinking, and sensitivity in order to stimulate them to recognize discriminations, inequalities, barriers, exploitations, and to act also from a professional point of view in favor of the overall wellness of humanity (Fernandez et al., 2018; Hooley et al., 2018).

As suggested by different authors, career interventions may support individual and collective reflections to promote collective empowerment and critical consciousness development to help people design their lives. It could be essential to stimulate young adults to be more oriented to undertake career activities that allow them to achieve their satisfaction and be less individualistic, to contribute to the realization of inclusive and sustainable social contexts (Blustein et al., 2020; García-Feijoo et al., 2020; Nota et al., 2020).

Given these considerations, we developed an online group career counseling intervention for young adults to support them in an inclusive and sustainable career design, including individual reflections, personalized activities, and group collective reflections and activities. Furthermore, the activity aims to promote positivity toward the future and career adaptability.

### Group career counseling for young adults

Career counseling was defined as a one-to-one or small group relationship between a client and a counselor to help the client integrate and apply an understanding of the self and of the environment to make the most appropriate career decisions and adjustments in this fast and changing world (Jiang et al., 2019).

The Life Design career counseling, based on Guichard’s Self-Construction Theory (2016) and Savickas’ Career Construction Theory (Savickas, 2012), provides a new perspective in helping clients in self-construction in every relevant life role by becoming aware of the salience of different life roles, defining priorities, identifying supports, cultivating resources, and engaging in different activities (Hirschi et al., 2020). Moreover, in the one-to-one or small group Life Design career counseling, it is also essential to help people reflect on the challenges that characterize our societies and to recreate a sense of their future by identifying what they can do to manage it (Guichard, 2018; Nota et al., 2020).

The group-based Life Designing counseling may be beneficial in terms of its potential social support (e.g., peer support), social skills trial, self-efficacy, and intrinsic motivation enhancement since sharing one’s own experience with the group may facilitate the construction of meaning in life (Barclay & Stoltz, 2016). Indeed, the success of peer groups in supporting people facing various difficulties has been proven in numerous contexts, also related to vocational guidance and career counseling (Pordelan et al., 2018), job performance (e.g., Becker-Beck et al., 2005; Zhang & Venkatesh, 2013), and job search. In this respect, success refers to improvements in the situation all individuals are currently struggling with or in the way they deal with it (Felgenhauer et al., 2019).

The Life Design paradigm on implementing career counseling in small groups can be considered particularly useful for many different reasons. It supports the movement from the private to the public sphere, from individual interview sessions to actions rooted in the collectivity and in the group, making them more relevant. This creates the opportunity to give life to more complex thoughts, bringing out the value of everybody’s participation in the educational and professional fields and in the construction of futures of quality (Nota et al., 2020; Savickas, 2015). Salvatore and Freda (2011) also supported the group's role, who asserted that a reflexive process could not be confined to individual minds.

In other words, in group counseling activities, each member of the relationship brings multiple viewpoints of the experience (i.e., one listens, considers, and elaborates), allowing the subject to provide new meaning to the experience and enhance a reflexive meaning construction of this experience.

To foster specific and personalized activities, Zainudin et al. (2020) suggest adding to group activities individual meetings as these activities can increase the effectiveness of group interventions. These individual meetings have the goal to highlight the individual needs and experiences within the context in which the individual lives. Furthermore, it is crucial to consider the pandemic situation that has ultimately led all counselors and career professionals worldwide to implement the online/cyber counseling process (Felgenhauer et al., 2019). In this scenario, Zainudin et al. (2020) asserted that online mixed individual and group career counseling appeared to be more effective and better used by the young (19–24 years old) and older adults (25–39 years old). Consequently, we developed online structured group career counseling that included one-on-one sessions to help young adults with their career planning.

### Online career counseling for young adults

The increasing use of technology in the field of counseling has led to the development of counselor competency standards and ethical guidelines for the use of technology. For example, in light of the COVID-19 global pandemic, the American Counseling Association (2020) organized resources and provided training sessions on ethical issues and considerations regarding the advantages and disadvantages of online counseling. Situmorang (2020) highlighted that the advantages are related to the absence of physical space, subsequently decreasing the requirement for travel, appointment-related concerns, and overhead costs. More prominent confidentiality, security, and privacy, and a subsequent improvement in a non-judgmental environment are also considered additional advantages. Despite the advantages of online counseling, concerns about time planning and the expertise of online counselors emerged during the pandemic period. Some critical features regarding online consuling were considered, such as technical preparation, adequate private computer access, and support for the untrained. Additionally, Situmorang (2020) also emphasizes the importance of considering the difficulties that can emerge in online counseling during the collection of non-verbal data normally obtained from direct observation of body language and other verbal and visual stimuli. In order to cope with these elements, Situmorang (2020) suggests that it could be important for an online counselor to develop relevant specialized information and to have a certain level of familiarity with online communication strategies to work with heterogeneous groups (such as the use of feelings to validate thoughts and emotions, the use of sharing slides, videos, and audio files).

Counselors who use online media should also sort rapidly, compose expressively, and be proficient at online communication and Web-browser administration. Wang et al. (2018) found that online counselors needed to be knowledgeable about the stage being used and multitask between technical/logistical and counseling activities components.

A few studies regarding online Life Design interventions with different samples have been published. Santilli et al. (2015) developed and validate the effectiveness of an online Life Design career intervention comprised of three 2-h sessions, each introduced with a video and specific activity for students (e.g., written exercises designed to help youth focus on their strengths, individualized interpretation of assessment results) held by computer. Specifically, the intervention aimed at helping students in their career construction, fostering career and life goal setting. Another example is provided by Pordelan et al. (2018), which developed five-session online counseling to promote students’ career development based on the Life Design paradigm. In all online counseling sessions, resources provided to clients included playing the content as audio, introducing online books, PowerPoint presentations, videos, short animations, online chats with a career counselor, and providing technical and educational support. Some authors have considered the need to carry out online career counseling interventions in this period since the global pandemic has brought a profound and widespread sense of uncertainty, fear, and loss (Autin et al., 2020). However, through practitioners' efforts that develop an intervention to promote collective advocacy, it is possible to shape a world where an inclusive, sustainable world and decent work exist. Through this, it is possible to highlight that the emerging sense of hope places the idea that our society can reinvent itself to be more just and equitable.

Referring to the above literature, we would develop and preliminarily assess the effectiveness of an online group career counseling intervention, divided into five sessions to incorporate the principles of the Life Design for an inclusive and sustainable future with personal and collective reflections. The activity was developed for young adults to engage in this time of crisis as an opportunity to help shape a world where decent, supportive, and dignified work is possible for all who want it.

### Aims of the study

A new and growing research area is starting to recognize that COVID, conceived as a macro event, may also influence young adults’ inclusion in the job market (Wanberg et al., 2020). Times of hardship and uncertainty contribute to a loss of control and increased difficulty in identifying future desires and goals because they are too focused on the present and the current concern (Bianchi, 2020). In raising awareness regarding unemployment during COVID-19, we referred to the research agenda developed by Blustein et al. (2020) that suggests understanding the experience of young adults in a participatory modality and developing and provide evidence-based interventions. To this end, this study aims to describe the development of online group career counseling intervention with a group of unemployed young adults implemented during the first COVID-19 lockdown in Italy (April 2020) and present the preliminary qualitative and quantitative analysis of its validity and effectiveness. Specifically, within the Life Design for an inclusive and sustainable future paradigm, we hypothesized that unemployed young adults engaged in the online group career counseling intervention would evidence, at the post-test, higher levels than the pre-test of: (a) career adaptability, a psycho-social construct that consists of four problem-solving and coping strategies or resources that help to cope with age-appropriate developmental tasks and transitions (concern, control, curiosity, and career confidence; Savickas & Porfeli, 2012); (b) resilience, the ability to quickly regain the energy and strength that are needed to take action when facing challenges that are threatening one's future plan (Masten & Obradović, 2006); (c) future orientation, that is the propensity to think possible futures related to behavioral flexibility and more effective planning for achieving goals (Schacter et al., 2008); (d) propensity to identify inclusive and sustainable actions for the future, able to support people in the construction of the future and guarantee their rights to participate in career contexts increasing a long-term vision that looks at individual, collective, and environmental well-being (Nota et al., 2020).

Furthermore, we hypothesized semantic differences in participants’ reflections between the three different group sessions and their thoughts about their future at the pre-and post-test. Specifically, lower correlations between participant’s ongoing self-reflection during group sessions were attended, with reflections gradually focusing on future intentions to undertake professional activities that contribute to realizing inclusive and sustainable social contexts and more positive thoughts about the future at post-test. Specifically, we hypothesized that the thoughts at the post-test would be more hopeful toward the future and characterized by an understanding of said future and by what individuals can do to manage it, as an indicator of the significant change in reflections and thoughts.

## Method

### Participants

In total, 12 young Italian adults interested in designing the future and looking for employment were involved, ten women and two men between the ages of 20 and 30 (*M* = 25.08; DS = 2.35) residing in Northeast Italy. Eight participants had a University degree, and four participants had a high school diploma. All of them were looking for a job: eight were looking for their first job, and four were looking for a job because of COVID-19 lockdown: they were not working and not receiving a salary.

### Measures

The following measures were administered to participants at the beginning and the end of the intervention:

***Career adapt-abilities scale-Italian form*** (CAAS-Italy for adults; Soresi, et al., 2012). It consists of 24 items, the same as in the CAAS-International Form 2.0 (Savickas & Porfeli, 2012). Participants responded to each item on a scale from 1 (*not strong*) to 5 (*very strong*). The 24 items combine into a total score indicating career adaptability. It consists of four subscales that measure the adaptability resources of Concern (e.g., “Preparing for the future”), Control (e.g., “Taking responsibility for my actions”), Curiosity (e.g., “Becoming curious about new opportunities”), and Confidence (e.g., “Overcoming obstacles”). A previous study conducted by Ginevra et al. (2017) showed that the total score and four subscales have reasonable internal consistency estimates (range 0.80 to 0.86) and a coherent multidimensional hierarchical structure in Italian adults, and the structure was similar to CAAS-International Form 2.0 (Savickas & Porfeli, 2012). For this study, Cronbach’s alpha for the four subscales were 0.83, 0.85, 0.87, and 0.80, respectively.

***Design my future*** (DMF; Di Maggio et al., 2016). It consists of 19 items on a scale from 1 (*It does not describe me at all*) to 5 (*It describes me very well*). It assesses Future orientation (11 items, e.g., “Thinking about the future excites me”); and Resilience (8 items, e.g., “Even under pressure, I'm able to concentrate, to think carefully about the purpose”). Previous analyses (see Di Maggio et al., 2016) demonstrated reliability and construct validity through exploratory and confirmatory factor analyses and measurement invariance across gender. Also, discriminant validity was confirmed with measures of career adaptability, optimism, pessimism, hope, and life satisfaction. For this study, Cronbach’s alpha for the two subscales were 0.88 and 0.80, respectively.

***Propensity for inclusive and sustainable actions for the future*** (Nota et al., 2020). The questionnaire comprises ten items with a scale from 1 (*It does not describe me at all*) to 5 (*It describes me very well*). It assesses the propensity to provide help and support and to spread inclusive feelings (e.g., “Standing up for those who may have suffered injustice and discrimination”), and the propensity to act sustainably and spread ideas of sustainability (e.g., “Bringing to mind the issues associated with the exploitation of natural resources and the fallout for all and sundry”). Previous analyses carried out by Ginevra et al. (2020) showed good psychometric properties for the scale with internal consistency estimates of 0.83 and 0.85. For this study, Cronbach’s alpha for the two subscales were 0.65 and 0.88, respectively.

***Ongoing self-reflection***. Considering Wilkins-Yel et al.’s (2020) study and Goodman et al.’s (2004) suggestions regarding the engagement of ongoing self-reflection as salient in counseling interventions, participants were invited to answer this question at the end of each online career counseling workshop section: “The most interesting point during the meeting was…”; “During the meeting, I was thinking about …". The answers to this question were examined while considering these categories that refer to the Life Design for an inclusive and sustainable future (Santilli et al., 2020): attention on the present, the self, and personal goals; attention to the future, relationships, and social challenges.

***Thoughts about the future*.** Considering Santilli et al.’s (2020) and Hartung’s (2015) studies, which suggested stimulating people’s reflections on their thoughts about the future, participants were invited to answer this question: “Considering this emergency, what do you think today about your future?”. The answers to this question were examined while considering the categories that refer to the Life Design for an inclusive and sustainable future (Santilli et al., 2020): attention on the present, the self, and personal goals; attention to the future, relationships, and social challenges.

***Social Validity***. To examine the social validity of the online group career counseling intervention, an item was administered at post-test to measure group participants’ overall satisfaction with the intervention. The item was based on Santilli et al.’s (2019) study: “How satisfied are you with this project in helping you think about the future considering also the coronavirus emergency period?”. Participants responded to the item using a 5-point Likert-type scale ranging from 1 (*not satisfied*) to 5 (*extremely satisfied*).

### Procedure

All participants were involved in the online group career counseling intervention through an employment agency of the community. The employment agency asked people who had approached during the pandemic period if they were interested in the activity that was also publicized through video ads on its social channels. To the people who showed interest in a career design, the intervention was advertised in detail, and after asking them if they wished to participate, and obtained their consent.

### Online group career counseling for young adults

The online group career counseling consists of five work sessions, two of which are individual (beginning and closing section) and have one hour each, while the remaining three are group sessions of 2 h each, bringing the total amount of work time to 8 h. Two online group career counseling interventions were conducted. Each online group counseling counted six randomly distributed participants.

#### Beginning

The online intervention began with about 1 h conducted individually through face-to-face online meetings in which a brief introduction of the member and the counselor was designed to build a connection among each participant. The counselor led participants first to describe what they hoped to get from experience. Subsequently, during the first meeting, the counselor tried to highlight the state experienced by the person looking for a job during the pandemic period. People narrated their training, work, and job search experiences in the past, their vision of the future, and their hopes, also initiating forms of reflection on what they have experienced. This individual meeting allowed to build a personalized action with each participant to pay attention to their narratability related to career adaptability, resilience, future orientation and contextual aspects associated with the situation experienced and the experienced barriers to starting to promote the development of new meanings to their difficulties.

#### During

The following 6 h were spent participating in an online group career counseling split into three sessions of two hours each on the topic of work. The online career counseling workshop was used to intentionally guide participants to deeply and personally engage with the topic of unemployment in this specific pandemic situation. Facilitators initiated the dialog by inviting participants to discuss the economic reasons behind unemployment, supplemented by the use of news and YouTube videos (Wilkins-Yel et al., 2020). Specifically, during the first session that was conducted in a small group (6 participants), the goal was to help people consider the contextual conditions related to precariousness, inequality, undignified work, and to stimulate them to analyze some of the situations experienced in the light of the aforementioned social phenomena. This reduces the perception that what is experienced is due solely to “individual responsibilities” and increasing their level of career concern, control and confidence. During the second session in a small group, the goal was to focus on human rights and workers’ rights, decent work, inclusion, and the value those themes can have in the next future and in professional life. This was done to stimulate a different and regenerative vision of the world, in line with what researchers and the best international institutions wish, for increasing their career resilience for decent and sustainable work. In the third and last session in a small group, the goal was to take into exam the idea of ‘future strivings', to stimulate the 'looking beyond the present, to identify challenges and threats that could be dealt with in one's future, to improve everyone's life, and to support their career curiosity and future orientation. This session provided an in-depth exchange, structured in a way that could give the individual the stimuli to start imagining their striving, intended as ‘mission possible’ (Nota et al., 2020). Based on Wilkins-Yel et al.’s (2020) suggestions, the counselor guided skill development by inviting participants to critically evaluate the sources of their information related to work and unemployment during COVID-19. After summarizing reflections that emerged during the meeting, counselor guided the activity at the end of the group session. Participants were asked to write what they most enjoyed about the workshop and what their thoughts were during the activity (“The most interesting point during the meeting was…”; “During the meeting I was thinking about…”). After writing these thoughts, the counselor anonymously read and processed written responses to all participants. This activity served as a way for young adults to normalize and validate each other’s experiences (Wilkins-Yel et al., 2020). The meeting closes with comments on the activities, underlining the positive aspects of the group, and reminding the date of the next meeting.

#### Closing

The last session of about 1 h was conducted individually through face-to-face online meetings. It was used to resume the description of participants’ striving/mission possible that can be carried out in the near future, connecting them to the emergencies/challenges that participants would like to improve and to reflect on one’s own strengths for a future design that can be helpful to realize these missions possible. Specifically, participants were helped identify occupations and professional activities related to their future agenda and possible training paths that can help them acquire knowledge and useful skills to perform them and handle the emergencies identified (Nota et al., 2020).

Furthermore, the counselor facilitated reflection on the intervention experience; specifically, participants were invited to think about how they planned to use this experience beyond the online intervention (Wilkins-Yel et al., 2020). Lastly, participants completed the measure with an online post-session evaluation.

### Data analysis

In order to evaluate the effectiveness of the career intervention, data were analyzed in different ways, including statistical significance tests and latent semantic analyses. The methods are described below.

### Statistical significance of the change

The effects of the online group career counseling intervention on career adaptability, future orientation and resilience, and the propensity to identify inclusive and sustainable actions for the future were evaluated using a nonparametric method (Wilcoxon signed-rank tests).

According to Cohen's ES (1988), the effect size was calculated for changes over time, applying the Wilcoxon Signed-Rank test (r) using the formula z/√N, where z is the test statistic, and N equals twice the number of individuals included in the respective analyses. According to Cohen's (1988) criteria, an effect size around 0.1 = low effect, 0.3 = medium effect, and 0.5 = large effect.

### Latent semantic analysis

The Latent Semantic Analysis (LSA) procedure was used to estimate the semantic differences in the reflections made by participants between the three different group sessions and in the thoughts about the future at pre- and post-test. For the goals of this study, we followed the holistic method applied by others (e.g., Dam & Kaufmann, 2008; Hartung & Santilli, 2018; Landauer, et al., 1998). The holistic method analyses the differences between participants’ ongoing self-reflection during the three online group career counseling and thoughts about the future at pre- and post-test. Lower correlations (< 0.05) between participants’ reflections in three different online group career counseling and thoughts about the future in pre-and post-test represent indicators of the significant change in reflections and thoughts.

## Results

### Statistical significance of change

The Wilcoxon test showed significant changes between pre-test and post-test on Career Adaptability, and specifically on Concern (*z* = -2.849, *p* = 0.004; *r* = 0.82), Control (*z* = -2.918, *p* = 0.004; *r* = 0.84), Curiosity (*z* = -1.966, *p* = 0.049; *r* = 0.56), and Confidence (*z* = -3.070, *p* = 0.002; *r* = 0.88). Furthermore, results highlighted changes in Resilience (*z* = -2.949, *p* = 0.003; *r* = 0.85), Future Orientation (*z* = -2.517, *p* = 0.012; *r* = 0.72), and Propensity to identify inclusive and sustainable actions for the future (*z* = -2.764, *p* = 0.006; *r* = 0.79). Participants involved in the online group career counseling presented at post-test higher level career adaptability, resilience, future orientation, and propensity to identify inclusive and sustainable actions for the future (see Table 1).

**Table 1:** Means and standard deviations of online group career counseling intervention at pre- and post-test

Measure	Pre-test		Post-test	
	*M*	SD	*M*	SD
Concern	20.83	2.21	25.50	1.98
Control	22.33	2.74	26.50	2.94
Curiosity	22.75	3.25	25.75	3.41
Confidence	22.42	1.93	26.75	2.14
Resilience	46.58	5.71	54.92	6.16
Future orientation	20.33	3.55	23.42	2.71
Propensity to identify inclusive and sustainable actions for the future	44.42	5.65	51.83	4.41

### Latent semantic analysis

Semantic differences in the reflections made by participants between the three different group sessions and in the thought about the future at pre-and post-test were calculated by LSA pairwise comparison (Landauer & Dumais, 1996). The LSA average correlation value related to the similarity between each three ongoing self-reflection is 0.42, specifically the 0.38 between the first and second ongoing self-reflection and 0.46 between the second and third ongoing self-reflection 0.46.

The level of similarity between the thoughts about the future at pre-and post-test was 0.42.

An example of ongoing reflections produced by a participant was: “…during the first meeting I was thinking about the concept of work, and I particularly appreciated the group atmosphere that was created because it did not make me feel alone in this difficult time”; “…during the second meeting I thought about the challenges of the future and the one we are experiencing, and I enjoyed reflecting on the relationship between Covid and the challenges that may arise in the future if we do not do something for the well-being of everyone”; “during the third meeting I had the opportunity to reflect on the fact that in the future to be successful professionals we should collaborate more and more with others and I liked to reconsidering work as a ‘mission possible’ to be accomplished with other professionals, that is important rethinking about the world and the concept of work with the future of our world.”

The thoughts about the future expressed by the participants at post-test t emphasized the attention on the present, the self, and personal goals and the attention to the future, relationships, and social challenges. For example, one participant stated at the pre-test: “…I think that in the future there will be many difficulties… the damages of this situation we will see in a few times from an economic point of view… also social, everyone fears the other… there will be more avoidance”. The same participants at the post-test stated: "I think that in the future there is a risk that more people may be exploited in the name of the crisis from a work point of view. I hope to open my enterprise in order to treat my employees with dignity and to create a good relationship between colleagues, respecting the rights not only of workers but also of the planet."

### Social validity

Overall, participants evaluated the online group career counseling as important for reflecting on specific dimensions of their careers and helping them cope with career future projects and transitions. A total of 75% (*N* = 9) of participants indicated that they were extremely satisfied with the overall program, and 25% (*N* = 3) were satisfied, none were neutral about it, none reported being a little satisfied, and none indicated that they were not at all satisfied with the program.

## Discussion

This study aimed to develop and examine the effectiveness of an online group career counseling intervention implemented during the COVID-19 with a sample of twelve young Italian adults interested in their career design. The goal of the online group career counseling intervention was to stimulate ideas of future planning that included an inclusive and sustainable idea about the future and the propensity to think about obtaining a decent job and a better future. In this way, we answered a call to conduct interventions providing tangible support in a period of health emergency and crisis for the economy and labor market, which can foster the process of collective empowerment and more complex contextual reflections development (Blustein et al., 2020).

Various methods were used to examine the effectiveness of the online group career counseling intervention. Specifically, the statistical significance test results showed that participants in the study experienced an overall increase in career adaptability, resilience, future orientation, and the propensity to identify inclusive and sustainable actions for the future.

The increased career adaptability (concern, control, curiosity, and confidence) may be due to the emphasis given to young adults to find their voices and identify their role in building a quality future for themselves and others. Specifically, participants may have experienced the possibility of positively project their professional future, of 'looking beyond the present, having a more active role in detecting challenges and threats that could be dealt with in their future to improve everyone's life (career control). Moreover, the online group career counseling intervention let young adults answer questions and face challenges about the future of humanity that required them to explore something that they recognized as being new to focus their attention on (career curiosity). By analyzing these challenges that they would have liked to face, the intervention allowed young adults to identify their ‘mission possible’, they are striving to achieve. This may have generated a sensation of self-efficacy that helps to successfully carry out the necessary actions to pursue said ‘mission possible’ (career confidence; Hirshi et al., 2015).

During the online group career counseling intervention, particular emphasis was given to the reflection on multiple potential scenarios and the analysis of the already experienced negative situations such as unemployment, work precariousness, inequality, and undignified work also in order to identify a new way to read them, to manage them, and to resist to the difficulties associated with them. This may explain participants' increased future orientation and resilience (Hite & McDonald, 2020; Nota et al., 2016; Santilli et al., 2017).

This intervention was also associated with an increased propensity to identify inclusive and sustainable actions for the future. As suggested by García-Feijoo et al. (2020), Nota et al. (2020), and Blustein et al. (2020), the collective reflections promoted during the intervention may have favored a greater propensity to get involved in order to contribute to the construction of inclusive and sustainable future contexts and the overall wellness of the humanity.

Therefore, having participants reflect on aspects dear to the Life Design inclusive and sustainable approach has stimulated new and richer reflections and thoughts about the future that are useful for a career design that is more in tune with our times. The aspects considered were human rights, decent work, decent life for everyone, especially those who experienced difficulties in the current labor market, such as young adults, and the processes that characterize the labor market and the social context, discriminations inequalities, barriers. As highlighted by Heberle et al. (2020), these results may derive from the socialization experiences and relationships developed during the online group career counseling intervention. In addition, these new reflections and thoughts are associated with the future intention to undertake professional activities that contribute to the realization of inclusive and sustainable social contexts.

Finally, the results showed that almost all participants perceived the online group career counseling intervention as satisfactory. This finding confirms that this intervention can adequately and positively respond to the requests of young adults who need career support in their career construction in times of need, such as the one associated with a global pandemic.

Overall, the results obtained show that the online group career counseling intervention, based on the Life Design for an inclusive and sustainable future, effectively supported young adults in designing a future, decent work, and life during the first COVID-19 lockdown in Italy (April 2020).

### Theoretical and practical implications

These findings have both theoretical and practical value. In terms of theoretical implications, findings advanced the literature on career interventions during the COVID-19 pandemic. Firstly, as Blustein et al. (2020) suggested, the study emphasizes the importance of developing career counseling activities to understand in participatory mode the work experience of young adults. Secondly, in line with Wanberg et al. (2020), the study highlights the importance of including in career counseling activities macro-level analyses of working conditions associated with indecent work (unemployment, precariousness, etc.). This can help reduce the perception that what is experienced is due solely to ‘individual responsibilities’ and leads to social reflections on the construction of professional futures that consider the well-being of humanity.

In terms of practical implications, the study provided the first evidence that online group career counseling intervention, which involves the stimulation of individual reflections through personalized activities and collective reflections through the use of collective activities, could be an effective strategy to involve young adults that experience difficulties during this pandemic period (Zainudin et al., 2020). The carried-out work suggests that it is possible to realize a career intervention with young adults able to move toward the future, getting away from the threats that humanity and Earth are suffering, such as the recent COVID-19 global pandemic, thinking about the challenges that they would like to face. This intervention will help outlining their desires, the things they have to learn and strengthen, the conditions to be encouraged and looked for, and the goals to be pursued with perseverance.

### Limitations and future research directions

Although our findings are encouraging, some limitations must be considered. Firstly, the sample size is relatively small; the gender composition is biased, with more women involved in the study and all participants live in Northeast Italy. Therefore, the generalizability of the results could be limited. Hopefully, future research should involve more men and participants from other Italian regions to verify the generalizability of the analyses reported in this study. Secondly, a control group has not been involved, and, in this sense, future research may verify the impact of the intervention on the participants compared to those who did not participate in it. Thirdly, it should also be specified that the effectiveness of the career intervention should not be limited to the examination of changes that occur only at the end of the career intervention. This implies that future research should also include 6- or 12-month follow-ups to verify whether young adults did pursue their goals or not.
